# Synthesis and Size Dependent Reflectance Study of Water Soluble SnS Nanoparticles

**DOI:** 10.3390/nano2010054

**Published:** 2012-01-16

**Authors:** Ying Xu, Najeh Al-Salim, Richard D. Tilley

**Affiliations:** 1 School of Chemical and Physical Sciences, MacDiarmid Institute of Advance Materials and Nanotechnology, Victoria University of Wellington, P.O. Box 600, Wellington 6011, New Zealand; Email: Sherry.Xu@vuw.ac.nz (Y.X); Email: richard.tilley@vuw.ac.nz (R.D.T); 2 Industrial Research, Ltd., P.O. Box 31-310, Lower Hutt 5010, New Zealand; Email: n.al-salim@irl.cri.nz

**Keywords:** tin sulfide, nanoparticles, triethanolamine, water soluble, diffuse reflectance

## Abstract

Near-monodispersed water soluble SnS nanoparticles in the diameter range of 3–6 nm are synthesized by a facile, solution based one-step approach using ethanolamine ligands. The optimal amount of triethanolamine is investigated. The effect of further heat treatment on the size of these SnS nanoparticles is discussed. Diffuse reflectance study of SnS nanoparticles agrees with predictions from quantum confinement model.

## 1. Introduction

Semiconductor nanoparticles are of great interest due to their unique size-dependent optical properties and potential applications in various nanoelectronic and optoelectronic devices [1,2,3,4,5,6]. Interest in the synthesis of group IV–VI semiconductor nanoparticles, such as PbS, SnSe and SnS, stems from their narrow band gaps and potential application in photovoltaics, near infrared detectors and biomedical applications [7,8,9,10,11,12]. These applications have stimulated the development of convenient synthetic methodologies to make monodisperse group IV–VI nanoparticles with narrow size distributions and good size-defined optical properties.

Unlike cadmium- and lead-containing nanomaterials, tin monosulfide, SnS, is considered to be a less-toxic material. SnS has both direct and indirect band gaps of 1.3 and 1.09 eV, respectively, and shows great promise in the field of infrared (IR) photoelectric and thermoelectric devices [13,14,15,16,17]. Various methods, such as solvothermal and hydrothermal, have been utilized to synthesise SnS nanostructures with different morphologies, such as nanosheets, nanorods [18], nanowires [19] and nanoflowers [18,20]. SnS has an orthorhombic layered crystal structure and can hardly grow into a zero-dimensional nanostructure, and therefore, it is a significant challenge to fabricate monodispersed SnS nanoparticles with a small enough size in a quantum confinement regime.

There are only a few reports that presented synthetic methodologies for forming SnS nanoparticles with a size of less than 10 nm. Hickey and co-workers demonstrated a method of forming SnS nanoparticles with size around 7 nm, using the air sensitive organometallic complex Sn[N[(SiCH_3_)_3_]_2_]_2_ as a tin precursor, thioacetamide as the sulphur prercursor, and trioctylphosphine (TOP) and oleic acid (OA) as surfactants [21]. More recently, Liu *et al.* reported the synthesis of around 6 nm SnS nanoparticles from SnCl_2_ and [(CH_3_)_3_Si]_2_S in oleylamine (OLA) [22], and Ning *et al.* prepared 5 nm SnS nanoparticles through the reaction of (Sn_6_O_4_(OH)_4_) with thioacetamide in the presence of OA and OLA [23]. However, in these syntheses the products were soluble only in organic solvents, which to some extent limits their use in the biomedical field [24].

In a previous communication [25], we reported a facile, room temperature synthesis to make SnS nanoparticles of a few nanometers in size. Herein, we report on the full synthetic methods, optical properties and the optimisation of some experimental parameters for the synthesis of SnS nanoparticles in solution using some simple starting materials, SnBr_2_ and Na_2_S, in the presence of ethanolamines, especially triethanolamine dissolved in ethylene glycol.

## 2. Results and Discussion

### 2.1. Synthesis and Properties

**Scheme 1:**
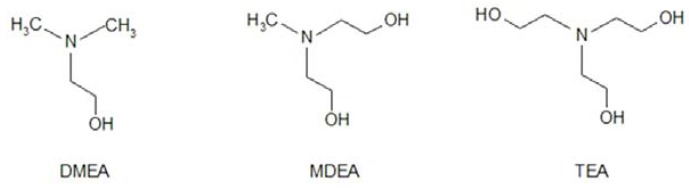
The chemical structure of DMEA, MDEA and TEA.

The SnS nanoparticles were synthesised through the reaction of the inorganic starting materials SnBr_2_ and Na_2_S in the presence of ethanolamines (EAs) in EG at ambient temperature. The ethanolamine ligands used had either one hydroxyl group (DMEA), two hydroxyl groups (MDEA) or three hydroxyl groups (TEA) (see Scheme 1).

We have previously shown [25] that the morphology of the EA-stabilized SnS nanoparticles were generally spherical, and in particular, TEA can produce highly monodispersed particles as compared to DMEA and MDEA. These results show that the SnS nanoparticle size and monodispersity can be controlled by the number of hydroxyl groups on the stabilizer. DMEA (one hydroxyl group) produced relatively bigger and less monodispersed nanoparticles than MDEA (two hydroxyl groups) which in turn formed bigger and less monodispersed nanoparticles than TEA (three hydroxyl groups). The greater monodispersity with increasing number of hydroxyl groups is more likely due to the ability of multiple hydroxyl groups to bind more strongly to the nanoparticles as they grow.

**Figure 1:**
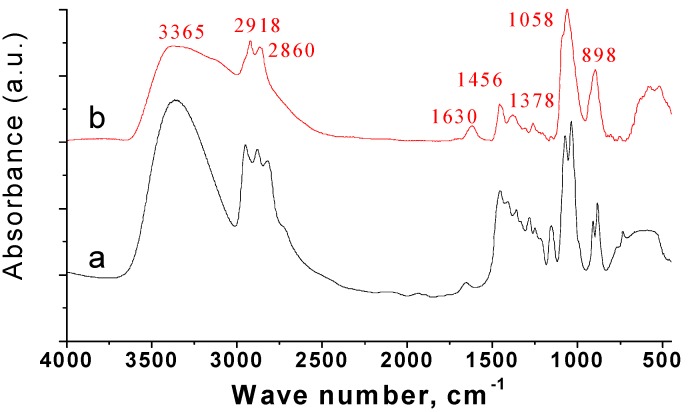
FT-IR spectra of (**a**) neat TEA and (**b**) TEA-capped SnS nanoparticles prepared at room temperature.

The bonding of triethanolamine to the surface of the SnS nanoparticles was confirmed by FT-IR spectroscopy (see Figure 1). The spectrum of the nanoparticles showed a significant absorption at 3,365 cm^−1^ with a shoulder, which are due to the strong O-H stretching vibrations and physisorbed and chemisorbed H_2_O [26]. Bands at 2,918 cm^−1^ and 2,860 cm^−1^ correspond to the C-H stretching vibration. A band at 1,630 cm^−1^ is due to H-O-H bending of H_2_O molecules. The bands in the region of 1,456–1,210 cm^−1^ are associated with vibrations of the C-C and C-H bonds. The C-O and C-N stretching vibrations were detected at 1,058 cm^−1^. The band at 898 cm^−1^ could be due to C-N, C-C, C-O and C-C-O vibrations.

**Figure 2:**
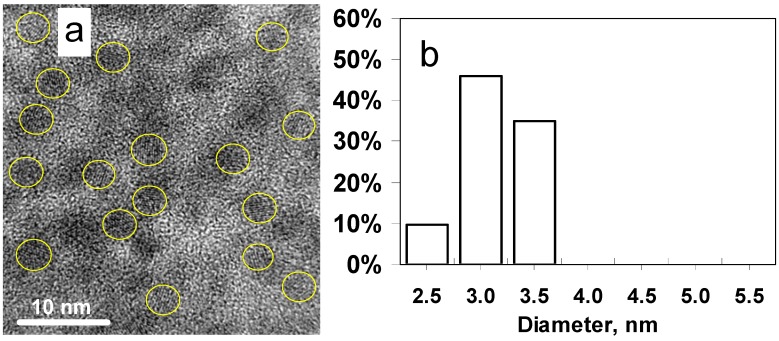
****(**a**) LRHTEM image of SnS nanoparticles obtained with 6 mL TEA; (**b**) Histogram of size distribution of SnS nanoparticles made with 6 mL TEA.

**Figure 3:**
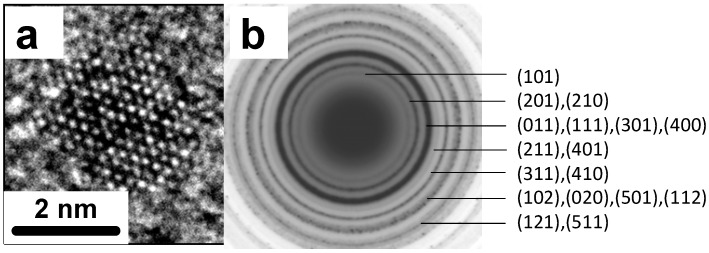
****(**a**) HRTEM image of a crystalline SnS nanoparticle obtained with 6 mL TEA; (**b**) Selected area electron diffraction (SAED) pattern of orthorhombic SnS NPs.

Figure 2a,b shows a TEM image of 3.0 nm SnS nanoparticles and the particle size distribution obtained with 6 mL TEA, respectively. The atomic lattice fringes are clearly observed demonstrating the highly crystalline nature of the nanoparticles. A HRTEM image of one SnS nanocrystal is shown in Figure 3a and the confirmation of the crystal structure of the TEA stabilised SnS obtained from SAED is shown in Figure 3b. The diffraction rings match well with the orthorhombic crystal structure adopted by SnS (Pbnm, a = 11.143 Å, b = 3.971 Å and c = 4.337 Å). The same crystal structure was obtained from MDEA/SnS and DMEA/SnS samples.

**Figure 4:**
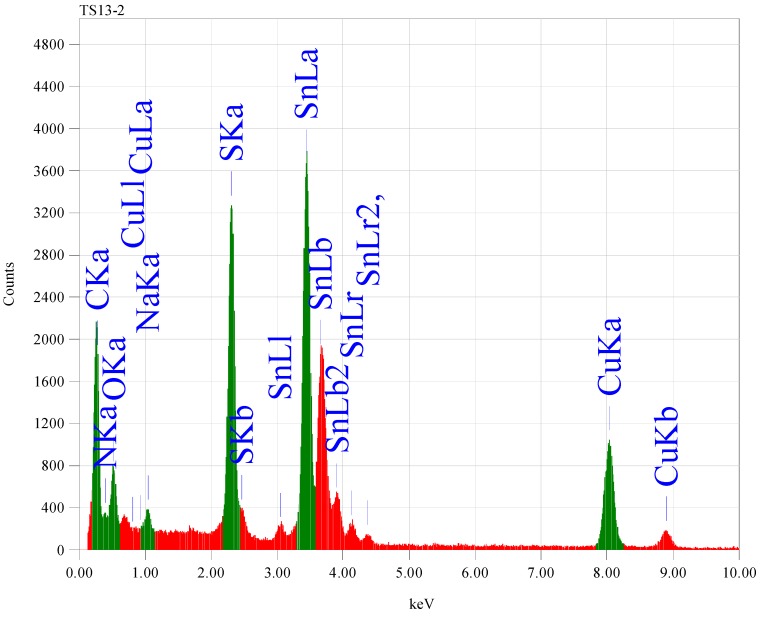
Energy dispersion X-ray spectrum (EDX) for SnS nanoparticles prepared in the presence of 6 mL TEA at room temperature.

The chemical composition of the as-prepared products using 6 mL TEA was examined by EDX analysis (Figure 4). The experimental data indicated that the Sn:S atomic ratio of ~1:1 is consistent with SnS formula.

### 2.2. Effect of the Amount of TEA

**Figure 5:**
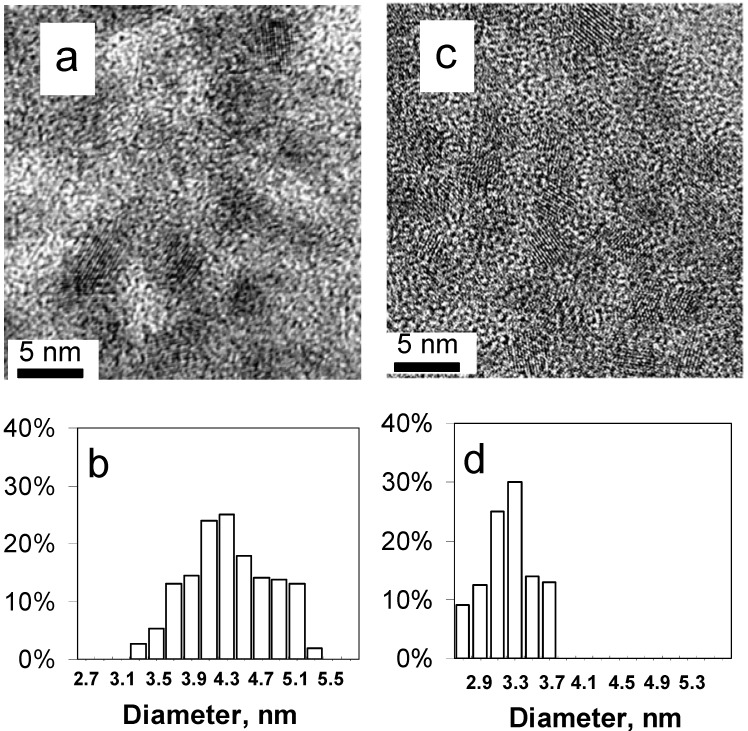
****TEM images of SnS nanoparticles synthesized in the presence of (**a**) 2 mL TEA; and (**c**) 4mL TEA at room temperature, respectively. Histograms of particle size distribution for (**b**) SnS nanoparticles made with 2 mL TEA; and (**d**) SnS nanoparticles made with 4 mL TEA.

In our previous work, a general method for making SnS nanoparticles in the presence of the ethanolamines TEA, MDEA and DMEA was described. Since TEA was found to be the most effective stabiliser among the studied ethanolamines in the formation of SnS nanoparticles, it is important to investigate the optimal amount of TEA required in the reaction. Previous reports showed that TEA can form chelate complexes with Sn^2+^ having a general formula [Sn(TEA)_n_]^2+^[27]. In the present reaction system TEA plays a dual role, first as a chelating ligand to the Sn^2+^ precursor and second as a stabilizer to the so produced SnS NPs. In this study, different TEA amounts (2, 4, 6, 8 and 10 mL) were investigated while maintaining other synthetic conditions unchanged. Figure 5a shows a TEM image of the SnS nanoparticles obtained in the presence of 2 mL TEA, in which the size of the SnS nanoparticles is 4.3 ± 1.0 nm as shown in Figure 5b. When 4 mL TEA was used, the SnS particle size was 3.3 ± 0.5 nm (Figure 5c,d). These can be compared with Figure 2a,b which shows the TEM image and the size distribution (3.0 ± 0.5 nm) of SnS nanoparticles respectively obtained in the presence of 6 mL TEA.

From the above data one can see that using 2 mL TEA gave slightly bigger and broader size distribution nanoparticles (4.3 ± 1.0 nm) than using 4 mL TEA (3.3 ± 0.5 nm) which in turn formed slightly bigger nanoparticles than using 6 mL TEA (3.0 ± 0.5 nm). Therefore, the size of SnS nanoparticles can be controlled by the amount of the stabilizing TEA ligand, such that the size of the nanoparticles decreased with increasing the TEA amount in the reaction system. Addition of TEA can control the growth of the SnS nuclei and form nearly uniform particles. It was noticed that using 8 mL TEA or more tended to highly stabilise the Sn^2+^ precursor complex, and therefore, slowed down the formation of SnS nanoparticles and gave less yield under the same conditions. Based on these results, 6 mL TEA per 0.2 mmol of SnBr_2_ could be considered as the optimum amount of this stabiliser for the synthesis of small and monodispersed SnS nanoparticles under the so used experimental conditions.

**Figure 6:**
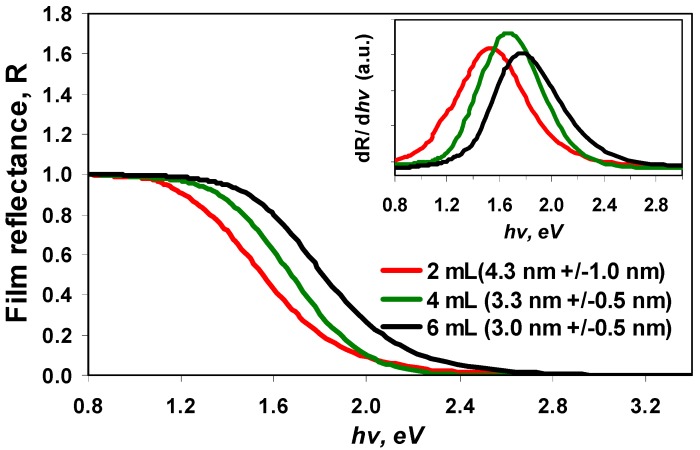
Reflectance spectra (normalised) of films that cast from different size nanoparticles made in the presence of 2, 4 and 6 mL TEA. Inset: The first derivative of film reflectance *versus* incident photon energy.

Figure 6 and the inset therein show the reflectance spectra of SnS nanoparticle of sizes 4.3, 3.3 and 3.0 nm prepared in the presence of 2 mL, 4 mL and 6 mL of TEA respectively [28]. The particles that were produced using 6 mL TEA showed a reflectivity edge at 1.76 eV. The larger nanoparticles produced using 4 mL and 2 mL TEA showed reflectivity edges that are red-shifted by ~0.1 eV and ~0.2 eV, respectively, compared to that of the SnS nanoparticles prepared using 6 mL TEA. The red shift of the reflectivity edge with increasing the nanoparticle size could be attributed to the quantum confinement effect.

### 2.3. Effect of Heat Treatment

**Figure 7:**
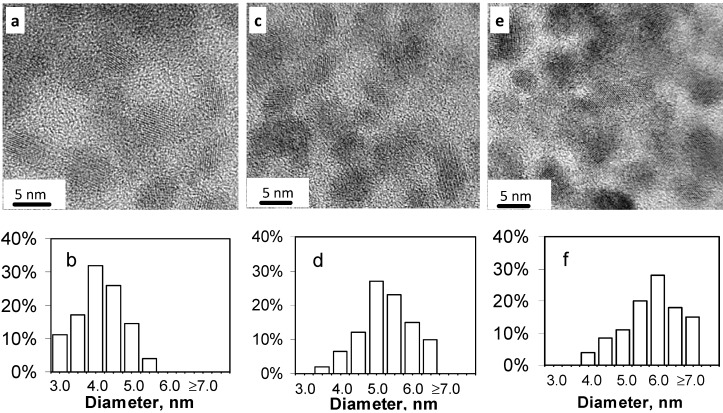
TEM images of SnS nanoparticles synthesized using 6 mL TEA at room temperature with further heat treatment at 100 °C for (**a**) 10 min, (**b**) 30 min, and (**c**) 60 min, respectively. The corresponding histograms of the size distribution for the SnS nanoparticles are shown in (**d**) 10 min, (**e**) 30 min, and (**f**) 60 min.

Previous research has shown that the average particle size and crystallinity of nanoparticles can be increased by either heat treatment at elevated temperature or longer time heating [29,30,31]. So, the effect of heat treatment on the properties of SnS nanoparticles is investigated here. SnS nanoparticles were first prepared at room temperature in the presence of 6 mL TEA, followed by further heating to 100 °C for 10, 30, and 60 min. TEM images showed that before heat treatment, the particles had predominantly spherical shape with an average size of 3.0 ± 0.5 nm, as shown in Figure 2a. After heat treatment, the particles became predominantly oval or have irregular shape with an increase in the average particle size depending on the heat treatment time (Figure 7). When the nanoparticles were heated for 10 minutes, the average size of the nanoparticles was 4.0 ± 1.0 nm. After heat treatment for 30 minutes, the nanoparticles grew to an average size of 5.1 ± 1.5 nm. By extending the heat treatment time to 60 minutes, the average size of the SnS nanoparticles reached to 6.0 ± 1.5 nm. The increase in the particle size may be explained by Ostwald ripening mechanism due to the heat treatment [32].

**Figure 8:**
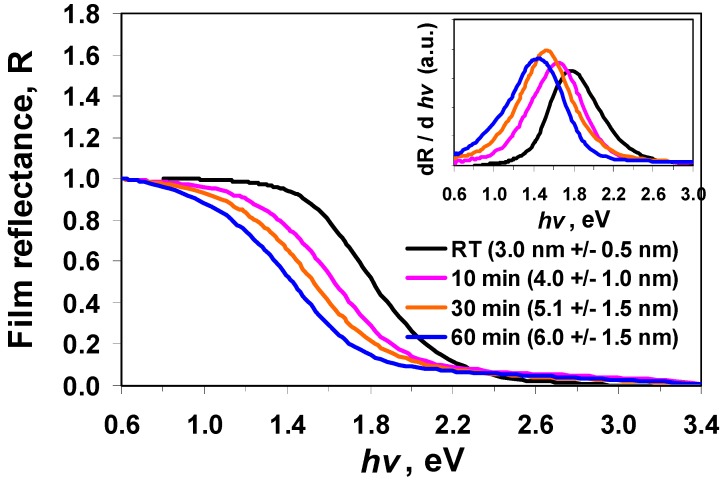
****Reflectance spectra (normalised) of SnS nanoparticle films that cast from particles made in the presence of 6 mL TEA at different heating time. Inset: The first derivative of film reflectance *versus* incident photon energy.

Figure 8 shows the diffuse reflectance spectra of the samples prepared with and without heat treatment. It can be seen from the derivative graphs in the inset that the reflectivity edge red-shifted from 1.76 to 1.45 eV by increasing the heat treatment time from 0 to 60 min. These results are significant as they illustrate the ability to control nanoparticle size in the quantum confinement regime leading to SnS nanoparticles exhibiting tunable optical properties. The inset in Figure 8 shows that the shape of the reflectivity derivative curves became broader after the particles were heated, which could be due to a wider size population caused by heating.

## 3. Experimental Section

### 3.1. Materials

All syntheses were performed under Argon. Tin(II) bromide (SnBr_2_ , anhydrous, 99.99%), sodium sulphide (Na_2_S, 98%) and ethylene glycol (EG, ≥99%) were purchased from Aldrich. Triethanolamine (TEA) was purchased from Pure Science. N-methyldiethanolamine (MDEA) and *N*,*N*-dimethylethanolamine (DMEA, 99%) were purchased from ACROS. All the chemicals were used as received without further purification. A 0.1 M Na_2_S solution was prepared by stirring an appropriate amount of Na_2_S in EG for 1 h at room temperature in an Argon atmosphere.

### 3.2. A Typical Synthesis of SnS Nanoparticles in the Presence of Triethanolamine

SnBr_2_ (0.056 g, 0.2 mmol) was dissolved in a mixture of 20 mL EG and 2, 4, 6, 8 or 10 mL TEA with vigorous stirring at room temperature under Argon flow. 2 mL of 0.1 M solution of Na_2_S in EG was added drop-wise over 10 min into the vigorously stirred SnBr_2_ solution. The resulting brown-coloured SnS nanoparticles were separated via centrifugation (50,000 rpm for 1–2 h) and washed repeatedly in ethanol for four times and stored in a desired solvent for subsequent morphology and optical properties measurement. A similar method was used to prepare MDEA and DMEA-tagged SnS NPs.

### 3.3. Heat Treatment

The synthesis was first carried out at room temperature in the presence of 6 mL TEA followed by heating at 100 °C for 10, 30 or 60 min.

### 3.4. Characterization Methods

Transmission Electron Microscopy (TEM), selected area electron diffraction (SAED) and energy dispersion X-ray spectroscopy (EDX) were recorded by a JEOL 2010 electron microscopy with an accelerating voltage of 200 kV. Samples were prepared by placing a drop of dilute, well dispersed colloidal solution onto a 200-mesh amorphous carbon-coated copper TEM grid purchased from ProSciTech. The software Gatan Digital Micrograph was used to capture and process images. Particle size distribution analyses were based on 120–150 nanoparticles.

Diffuse reflectance spectra were recorded on a High Accuracy Reference Spectrophotometer at IRL [26] in the wavelength range from 250 nm to 2,500 nm. The reflectivity measurements were made on drop-cast films of as-prepared nanoparticles deposited on glass substrates.

Fourier Transform Infrared (FT-IR) spectra were recorded using PerkinElmer Spectrum One FT-IR spectrometer with wave number range of 4,000–400 cm^−1^. Pellets for FT-IR measurement were prepared by mixing the dried samples (after purification) with spectroscopic grade KBr powder. Equal mass of KBr was used as a reference.

## 4. Conclusions

The synthesis of monodispersed colloidal SnS nanoparticles with sizes of a few nanometers were successfully demonstrated using ethanolamine ligands. The results show that ethanolamines, particularly TEA, are a class of ligands that can be used as stabilisers for SnS nanoparticles and other metal chalcogenide nanoparticles, such as SnTe [33].

The size of the resulting SnS nanoparticles can be tuned by changing the reaction parameters. Surfactants were shown to be the key factor in the size control of SnS nanoparticles. Due to the higher chelating effect of the three hydroxyl groups, TEA molecule proved to give the best particle size control compared to MDEA and DMEA. The optimal amount of TEA in the reaction system has been investigated. Heat treatment of the nanoparticles led to an increase in the particle size. Diffuse reflectance study of the SnS nanoparticles showed a size dependent effect on the reflectivity which may due to the quantum confinement. These results further unlock the potential of SnS materials for photocatalysts and solid state batteries [34,35,36,37,38].
